# In-hospital postoperative opioid use and its trends in neurosurgery between 2007 and 2018

**DOI:** 10.1007/s00701-021-05021-9

**Published:** 2021-10-18

**Authors:** Ilari Rautalin, Miia Kallio, Miikka Korja

**Affiliations:** 1 Department of Neurosurgery, University of Helsinki and Helsinki University Hospital, P.O. Box 266, 00029 Helsinki, Finland; 2 HUS Pharmacy, Hospital Pharmacy of Helsinki University Hospital (HUS), P.O. Box 440, 00029 Helsinki, Finland

**Keywords:** Analgesics, Opioid, Neurosurgery, Opioid epidemic

## Abstract

**Background:**

Postoperative opioid use plays an important role in the global opioid crisis, but little is known about in-hospital opioid use trends of large surgical units. We investigated whether postoperative in-hospital opioid consumption changed in a large academic neurosurgical unit between 2007 and 2018.

**Methods:**

We extracted the data of consumed opioids in the neurosurgical intensive care unit and two bed wards between 2007 and 2018. Besides overall consumption, we analyzed the trends for weak (tramadol and codeine), strong, and the most commonly used opioids. The use of various opioids was standardized using the defined daily doses (DDDs) of each opioid agent. A linear regression analysis was performed to estimate annual treatment day-adjusted changes with 95% confidence intervals.

**Results:**

Overall, 121 361 opioid DDDs were consumed during the 196 199 treatment days. Oxycodone was the most commonly used postoperative opioid (49% of all used opioids) in neurosurgery. In the bed wards, the use of oral oxycodone increased 375% (on average 13% (9–17%) per year), and the use of transdermal buprenorphine 930% (on average 26% (9–45%) per year) over the 12-year period. Despite the increased use of strong opioids in the bed wards (on average 3% (1–4%) per year), overall opioid use decreased 39% (on average 6% (4–7%) per year) between 2007 and 2018.

**Conclusions:**

Due to the increase of strong opioid use in the surgical bed wards, we encourage other large teaching hospitals and surgical units to investigate whether their opioid use trends are similarly worrisome and whether the opioid consumption changes in the hospital setting are transferred to opioid use patterns or opioid-related harms after discharge.

**Supplementary Information:**

The online version contains supplementary material available at 10.1007/s00701-021-05021-9.

## Introduction

Opioids are typically used to manage postoperative pain. In fact, the World Health Organization (WHO) considers opioids as essential medicines for pain and palliative care management [39]. Unfortunately, opioids have higher risks for abuse, addiction, and overuse than other analgesics [33]. During the recent decades, the incidence of opioid abuse, addiction, and overdose-related deaths has increased rapidly in high-income countries [30]. According to the latest United Nations’ drug report, opioids were responsible for two-thirds of the 585 000 overdose-related drug deaths in 2017. Especially in the United States (US), the misuse of opioids has reached the level of a national crisis, impacting not only public health but also social and economic welfare [18, 37]. Since opioid consumption in the hospital environment can substantially increase the risk of continued opioid use [3, 15, 17, 22], hospital-based opioid use policies are likely to play a crucial role in the global opioid epidemic.

People are often exposed to opioids for the first time in hospitals, particularly after surgery. Although several studies have assessed the trends of prescription opioid consumption outside hospitals and after hospital discharge [2, 16, 20, 21, 34, 35], surprisingly little is known about the trends of in-hospital postoperative opioid use of large surgical units. Moreover, since there are no previous studies that have assessed the in-hospital opioid use and its time-dependent trends in neurosurgery, we aimed to investigate whether in-hospital opioid use patterns have changed in a large European neurosurgical unit between 2007 and 2018. To evaluate whether consumption trends follows the changes of operation types, we also reported the case mix changes over time. We hypothesized that despite the proportion of mini-invasive operations (e.g., endovascular procedures) has increased in our neurosurgical unit during the last 12 years, the consumption of the most commonly misused opioid agents (e.g., oxycodone) has not been decreased accordingly.

## Materials and methods

### Ethical consideration

The study was conducted in line with the Declaration of Helsinki and received an approval (reference number: HUS/157/2020; date of approval: 14/09/2020) from the institutional review board of Helsinki University Hospital (HUH). According to Finnish legislation, informed patient consents are not required for retrospective and register-based studies with no patient identifiable information.

### Study hospital

HUH is the largest hospital in Finland, with over 90 000 operations performed annually. The HUH neurosurgical unit is the largest of the five in Finland, providing tertiary health care for the whole catchment area of approximately 2.2 million inhabitants (40% of the Finnish population). The neurosurgical department performs roughly 4 000 annual procedures. Its neurosurgical intensive care unit (ICU) has 16 beds, and the two neurosurgical wards have around 50 beds, depending on the year. In terms of pain management policies, the postoperative analgesia of both ICU and bed wards is usually managed together by neuroanesthesiologists and nurses. The specialized pain management team is consulted infrequently, most commonly in cases when a patient has a complex preoperative pain medication history. In bed wards, no department-specific guidelines for standardized postoperative analgesia have been in use, but the postoperative pain management is rather based on the experience of neurosurgeons, neuroanesthesiologists, and neurosurgical nurses. In the ICU, the pain management policies were standardized in 2015, and they follow the guidelines published by the American College of Critical Care Medicine [1, 14]. To the best of our knowledge, no other major policy changes have happened in our study unit during the study period.

### Opioid data

To estimate trends and patterns in opioid consumption, we extracted the opioid consumption from the enterprise resource planning (ERP) system of the HUH pharmacy. The ERP system defines opioid consumption by every ward and ICU using the anatomical therapeutic chemical (ATC) codes [28]. Along with the opioid agents classified to the general opioid class (code N02A), we also extracted consumption data on the opioids classified for use in opioid replacement therapy (code N07BC) and general anesthesia (code N01A). To standardize the potency of each opioid agent, all consumption levels were measured using defined daily doses (DDD) established by the WHO [38]. Specifically, yearly consumption of each opioid (obtained by multiplying the amount of drug per item by the number of issued items) was divided by an agent-specific conversion factor (detailed conversion factors, i.e., DDDs are described in Online Resource 1). Since the DDD for combined products are established by the number of items, the yearly consumption of combination products (e.g., codeine and paracetamol) was defined by the number of issued tablets. Moreover, as specific DDDs have not been defined for general anesthesia drugs (i.e., intravenous fentanyl and alfentanil in our case), we calculated the DDDs for these products based on their relative effects compared to oral morphine and transdermally administered fentanyl [29]. We classified the route of administration as either (1) transdermal (patch), (2) injection (intravenous (IV), intramuscular (IM), or subcutaneous (SC)), or (3) oral. In transdermal administration, we approximated that a patch of buprenorphine had been used for 7 days (i.e., 168 h) and a patch of fentanyl for 3 days (i.e., 72 h). In addition to the general trends in the ICU and bed wards, we also reported consumption trends for weak (tramadol and codeine) and strong (other opioid agents) opioids, as well as for the most commonly used opioids (> 1% of the overall opioid consumption). Opioid consumption was defined by subtracting inventory losses of expired opioids (not used before expiring) from the orders.

### Caseload, case mix, and study period

To consider the changes of yearly caseload in our neurosurgical unit, we divided the annual DDD-adjusted in-hospital opioid consumption by the number of treatment days during the year. The annual consumption levels were reported per 100 bed days as recommended by the WHO [38]. As treatment days at HUH have been registered to an electronic hospital-based register (Cressida™, CGI) since mid-2006, we studied the opioid consumption in the neurosurgical ICU and wards between 2007 and 2018. In terms of case mix and its time-dependent trends, we extracted the number and type of annual operations by utilizing the operation codes, registered to an electronic operating theatre management solution of HUH (Centricity™ Opera (GE Healthcare)). Case mix and its trends were evaluated by the ten most common operation types. As the operation codes have been used consistently in our study unit since 2012, we evaluated the annual case mix changes between 2012 and 2018. In addition, we compared the distribution of operation types between the first (2012–2014) and the last 3 years (2016–2018).

### Statistical analyses

We used scatter plots and fitted regression lines with 95% confidence intervals (CIs) to illustrate the consumption trends. In order to estimate average annual changes and trends of linearity, we used a linear regression model to calculate yearly estimates as percentages with 95% confidence intervals (CIs). All statistical analyses were performed by Stata version 16.1 (Stata Corp, College Station, TX).

## Results

### Treatment days and case mix

Between 2007 and 2018, there were 196 199 treatment days, of which 75.9% occurred in the bed wards and 24.1% in the ICU. Treatment days decreased by 1.4% (95% CIs 0.8–2.1%) per year in the bed wards, whereas no such decrease was observed in the ICU (Online Resource 2). Regarding case mix, 23 158 operations were performed between 2012 and 2018. No significant change occurred in the number of annual operations (Online Resource 3). Spinal fusions (17% of all operations), intracranial tumor surgeries (13% of all operations), and spinal decompressions (10% of all operations) were the three most common operation types between 2012 and 2018. The number of intracranial tumor surgeries decreased by 5.7% (3.5–8.0%) per year, whereas the number of spinal decompressions increased by 4.4% (2.6–6.3%) per year. No significant change was found in the annual number of spinal fusions. The most significant decrease was found in the number of cerebrovascular surgeries (14.9% (10.3–19.2%) per year), whereas the number of endovascular operations increased the most (27.5% (18.7–36.9%) per year) (Online Resource 3).

### Overall opioid use

**Fig. 1:**
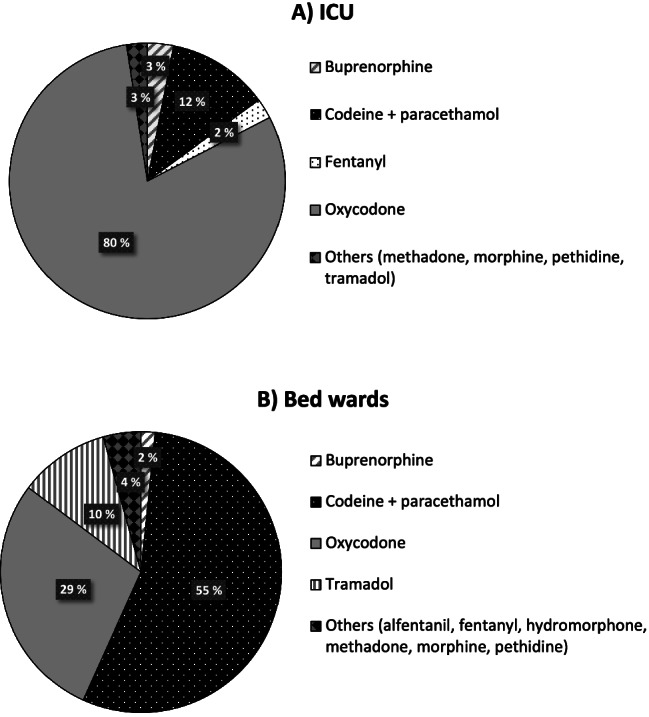
Proportions of consumed opioid agents in the **a** ICU and **b** bed wards between 2007 and 2018

Between 2007 and 2018, 121 361 DDDs (0.6 DDD per treatment day) were consumed in the bed wards and ICU. Of these, 60.6% and 39.4% were used in the bed wards and ICU, respectively. By treatment days, opioid use was two times higher in the ICU (1.0 DDD per treatment day) than in the bed wards (0.5 DDD per treatment day). When comparing the opioid-specific DDDs, oxycodone—which comprised 49% of all used opioids—was the most commonly used opioid in the ICU and the second most commonly used opioid in the bed wards. Weak opioid agents, namely codeine and tramadol, comprised over half of the used opioids in the bed wards, but less than 14% in the ICU (Fig. 1). Over 84% of the consumed opioids were administered orally in the bed wards, whereas over 87% of the used opioids in the ICU were injections (IV/IM/SC) (Online Resource 4).

### Time trends in opioid use

**Fig. 2:**
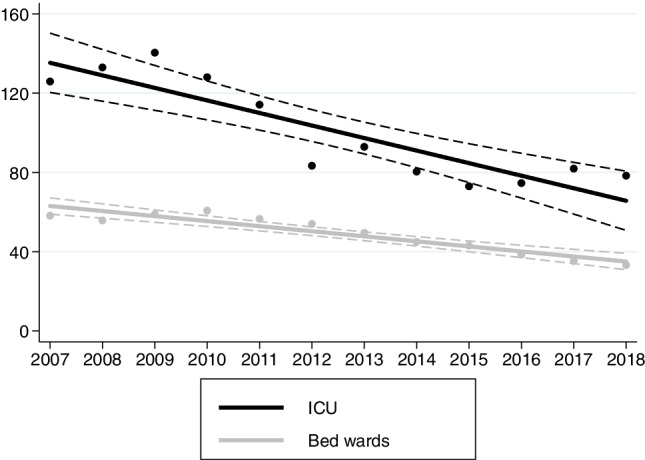
Trends of overall opioid use in the ICU and bed wards. The Y-axis describes adjusted yearly consumption rates (DDDs/100 bed days). Solid lines indicate fitted regression values; dashed lines indicate 95% confidence intervals

**Fig. 3:**
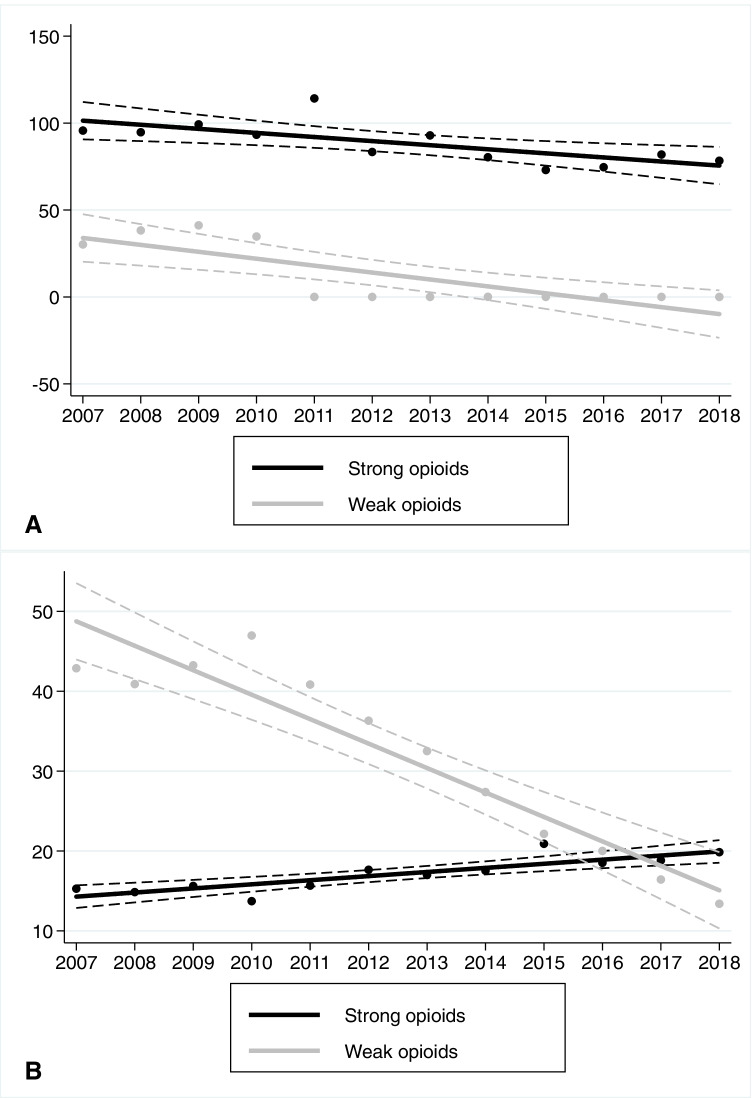
Trends of strong (black line) and weak (gray line) opioid consumption in the **a** ICU and **b** bed wards between 2007 and 2018. The Y-axis describes adjusted yearly consumption rates (DDDs/100 bed days). Solid lines indicate fitted regression values; dashed lines indicate 95% confidence intervals

Between 2007 and 2018, DDD-adjusted opioid consumption decreased by 6% (95% CI 4–7%) per year. This decrease was evident both in the ICU and bed wards (Fig. 2). The use of weak opioids (codeine and tramadol) decreased by 10% (8–13%) per year in the bed wards, and almost completely ceased in the ICU in 2011 (Fig. 3). In terms of strong opioids (i.e., alfentanil, buprenorphine, fentanyl, hydromorphone, methadone, morphine, oxycodone, and pethidine), we found a 3% (1–4%) annual decrease in the ICU. On the contrary, the use of strong opioids increased annually by 3% (1–4%) in the bed wards (Fig. 3). When assessing changes in opioid administration routes, the only increasing trend—which was 11% (3–20%) per year—was the use of transdermal opioids in the bed wards. Otherwise, the use of oral, injected, and transdermal opioids in the ICU decreased over time (Online Resource 5).

### Opioid-specific trends in the ICU

**Fig. 4:**
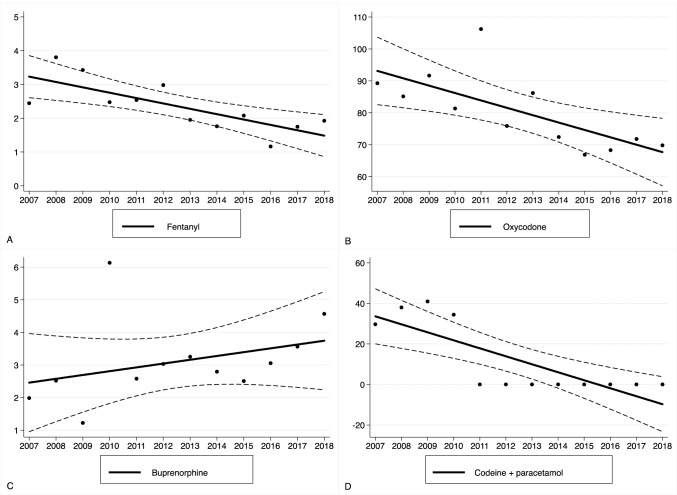
Trends in opioid agent-specific consumption in the ICU between 2007 and 2018. The Y-axis describes adjusted yearly consumption rates (DDDs/100 bed days). Solid lines indicate fitted regression values; dashed lines indicate 95% confidence intervals. **a** Fentanyl (injected). **b** Oxycodone (injected). **c** Buprenorphine (injected). **d** Codeine + paracetamol. Since the true consumption of codeine + paracetamol ended almost completely in 2011 (black dots), the fitted regression line (illustrating the trend of consumption) has negative values onwards 2015

Figure 4 illustrates the DDD-adjusted trends of the most commonly used (> 1% of all opioids) opioid agents in the ICU. The consumption of injected oxycodone and fentanyl decreased linearly, with annual estimates of 3% (1–5%) and 7% (3–10%), respectively. On the contrary, the use of injected buprenorphine increased by 5% per year, but this trend did not reach statistical significance (95% CI − 2 to 13%). As mentioned above, the use of oral codeine and paracetamol ended almost completely in 2011. Since oral and transdermal opioids were used very infrequently in the ICU, we did not assess agent-specific trends for orally or transdermally administered opioids.

### Opioid-specific trends in the bed wards

**Fig. 5:**
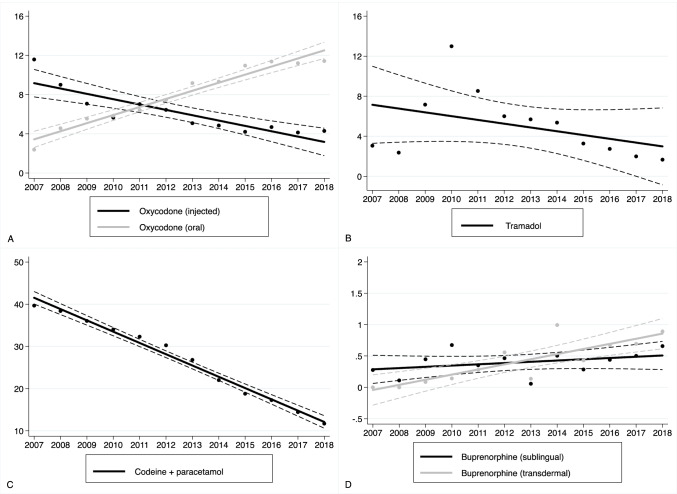
Trends in opioid agent-specific consumption in bed wards between 2007 and 2018. The Y-axis describes adjusted yearly consumption rates (DDDs/100 bed days). Solid lines indicate fitted regression values; dashed lines indicate 95% confidence intervals. **a** Oxycodone (oral and injected). **b** Tramadol (oral). **c** Codeine (+ paracetamol) (oral). **d** Buprenorphine

Figure 5 depicts the DDD-adjusted trends of opioid use in the bed wards. The most frequently used strong opioid agent was oxycodone, the use of which increased by 2% (1–3%) per year. Specifically, the use of injected oxycodone (which represents 45% of the overall oxycodone DDD-consumption in the bed wards) decreased by 8% (6–10%) per year. The use of oral oxycodone (which represents 55% of the overall oxycodone DDD consumption in the bed wards) increased by 13% (9–17%) per year. Moreover, we found that the use of transdermal buprenorphine patches increased by 26% (9–45%) per year. However, the use of transdermal buprenorphine comprised only 2.3% (in DDDs) of all strong opioids used in the wards. We found that the use of sublingual buprenorphine tablets increased during the study period, but this trend did not reach statistical significance. In terms of weak opioid agents, the use of oral codeine and paracetamol decreased by 10% (9–12%) per year. The decrease in oral tramadol use did not reach statistical significance (8% (− 3 to 17%)).

## Discussion

In light of the globally increasing opioid epidemic and its potential hospital-based background, our study provides many noteworthy findings about the trends of in-hospital opioid use in a large and academic surgical unit. Primarily, the overall opioid consumption decreased by 39% during the 12-year study period. This decreasing trend was observed both in the ICU and the bed wards and applied to both injected and oral opioids. The decrease of overall opioid use may at least partly be explained by the reported case mix changes. In other words, the transition from more invasive craniotomies such as cerebrovascular surgeries to so-called mini-invasive operations such as endovascular procedures may have contributed to the decrease in opioid consumption. However, most of this decrease was attributed to the drastic 82% decline (on average 16% (14–19%) per year) in the overall use of weak opioids, both in the bed wards and the ICU, whereas the overall use of strong opioids decreased by only 12% (on average 2% (0–3%) per year) during the 12 years.

Despite the decreasing number of craniotomies, our study showed that the oral oxycodone use increased by 375% in the bed wards during the study period. For example, oral oxycodone comprised 4% of all opioids used in the bed wards (in DDDs) in 2007; this figure increased to 34% by the end of the study period (2018). Since over-prescription of oral oxycodone is one of the key contributors to the global opioid crisis [31], it is possible that postoperative overuse of strong opioids during the hospital stay is the beginning of opioid misuse or even addiction for some patients. Interestingly, the increased oxycodone use was evident only in the bed wards, whereas the use of oxycodone in the ICU decreased over time. One possible explanation for the difference could relate to the established guideline for standardized pain management in the ICU since 2015. On the other hand, since the decreasing trend in the ICU occurred already before 2015, other factors are likely attributing to these differences, too. In the light of the case mix changes, the difference may also relate partly to the increased number of spinal surgeries, since the patients undergoing spinal surgeries tend to receive strong opioids particularly in the bed wards, as these patients are rarely treated in the ICU. However, since the number of the most common spinal surgeries (spinal fusions, decompressions and discectomies) increased only by 3% between the first (2012–2014) and the last 3 years (2016–2018), the increased number of spinal surgeries unlikely explains the increased use of oxycodone and other strong opioids in our bed wards. In addition to the vast increase in the oxycodone use, we found that the use of buprenorphine patches increased by 930% between 2009 and 2018. Before 2009, buprenorphine patches were not used at all. Even though buprenorphine patches comprised only 3% (in DDDs) of all used opioids in the bed wards in 2018, the observed trend needs to be carefully followed, especially since buprenorphine has become the most commonly abused opioid in Finland [7, 11].

Of all opioids, the consumption of oxycodone and buprenorphine has increased most significantly; currently, they account for the most commonly consumed strong opioids in Finland [9]. Furthermore, overdose-related deaths seem to follow the opioid consumption trends in Finland: 0.5% of all deaths in 2010–2011 had a history of opioid abuse, 85% of which involved buprenorphine abuse [11]. In comparison, the same amount of people died from traffic accidents as from opioid use in Finland during the same time period. In many countries, including Finland, the mean age of drug-related deaths is below 40 years [23]. Hence, the opioid epidemic and related deaths cause enormous losses of productive life years, and a general public health burden. In our study, the use of strong opioids in bed wards was relatively frequent. In Finland, the use of oxycodone and buprenorphine have increased by 63% and 820% between 2007 and 2018, respectively (Online Resource 6). Accordingly, in our study, these figures were 13% and 408% in the bed wards, respectively. However, the increase in the bed ward use can be at least partly explained by our step-down unit, which is part of one of the two bed wards. In fact, the bed ward with the step-down unit consumed slightly more injected and strong opioids (Online Resource 7). Nevertheless, the overall opioid consumption was still more common in the regular surgical ward (Online Resource 7). In terms of the opioid consumption trends, no significant differences were found between these two bed wards.

In the early twenty-first century, the increased marketing and use of oral oxycodone in North America has been attributed to initiating the global opioid epidemic [31]. In fact, oxycodone consumption increased more than five-fold in the US between 1999 and 2011; similarly, opioid-related mortality showed a four-fold increase over the same time period [19]. After 2010, prescription opioid use has fortunately decreased in both the US and Canada due to improved drug management policies [4, 10]. Nevertheless, opioid-related mortality and misuse rates have continued to increase due to the shift from prescription opioids to the illicitly manufactured fentanyl derivatives and heroin [24, 26]. Even though the opioid epidemic has escalated the most in North America, several studies [6, 12, 13, 25, 27, 36] have reported that opioid prescription rates, abuse, addiction, and overdose-related deaths are also increasing in many European countries such as Germany, France, the UK, and the Netherlands. Unlike North America, the prescription and misuse of opioids are increasing in Europe; the increased use of strong opioids is particularly alarming [6, 12, 13]. Moreover, although the estimated use of illegal fentanyl derivatives and heroin is still relatively infrequent in Europe, their confiscation rates have increased remarkably during recent years [8]. Fortunately, the European drug marketing system does not encourage individual physicians to prescribe excessive opioids as is the practice in North America [32], which was one of the main reasons for opioid overuse in both the US and Canada.

Whether our findings from a single surgical unit can be generalized more widely is debatable. However, there are a few reasons why our findings may mirror the situations in other surgical units. First, HUH is one of the largest academic hospitals in Europe; numerous international neurosurgeons and neurosurgical residents visit the neurosurgical unit every year. Therefore, clinical practices at HUH may be transferred to other neurosurgical units along with national and international visitors. Second, HUH’s neurosurgical unit educates a great number of neuroanesthesiologists, nurse staff, and nurse students who have a critical role in the postoperative analgesia management. Thus, they may have assimilated and spread the neurosurgical postoperative pain management protocols. Taking these aspects into account, focusing only on the opioid prescription trends and policies outside the hospital environment without consideration of the in-hospital practices may not provide a clear and comprehensive picture about the factors contributing to the increasing opioid crisis.

Our study may have at least one significant advantage. Despite several studies have assessed the trends of prescription opioid consumption after the hospital discharge [2, 16, 20, 21, 34, 35], to the best of our knowledge, no previous study has evaluated the trends of in-hospital postoperative opioid consumption. We believe that the origin of opioid misuse lies partly in the surgical units, and therefore studying the opioid use policies in a hospital environment should gain more attention in the future. If people are introduced to strong opioids during the early postoperative care (i.e., during the hospitalization period), it is more likely that these drugs will be prescribed also after discharge [5].

Our study has also limitations. First, even though we were able to consider the annual case load and case mix changes, we had no patient-level data, and therefore, we could not investigate whether opioid consumption trends linked to a specific patient group or to a certain operation type. In other words, our institutional-level data did not enable us to investigate for example whether opioid use changed only in men/women or in patients who underwent spinal surgeries during the study period. For the same reason, we were not able to investigate whether patients’ pain experience changed during the study period. If the postoperative pain control has improved without any evidence of opioid-related side-effects, the detected increase in opioid consumption may in fact be justifiable. Second, we have no data about the preoperative opioid use patterns during the study period. Therefore, it is possible that the preoperative opioid use especially among the patients with spinal problems has also increased during the study period, and this may have led to the increased in-hospital use, too. On the other hand, strong opioids (such as oxycodone or fentanyl) are rarely used preoperatively. Third, since we utilized the department-wide consumption data, we did not have the data on actual opioid prescriptions in the hospital or at/after discharge. Therefore, we were not able to study whether the opioid consumption changes related to the prescription policy changes by subspeciality (e.g., neurosurgeons or neuroanesthesiologists), or whether these changes in the hospital setting are transferred to opioid use patterns or opioid-related harms after discharge. However, it is known that opioid use within the last 24 h before hospital discharge is associated with the number of opioid prescriptions [5]. This considered, early discharge after surgery may predispose patients to overuse of opioids outside the hospital environment, which may in turn increase the risk of opioid abuse. Nevertheless, future studies are needed not only to evaluate whether similar trends can be found in other large teaching hospitals and surgical units, but also whether our observations may be explained by changes in preoperative pain management policies, and whether these observations are related to patients’ in-hospital pain experience and to opioid prescriptions, abuse, addiction, and overdose-related deaths after discharge.

In conclusion, despite the overall opioid consumption decrease in neurosurgery, we noted a vast increase in the use of oral oxycodone and transdermal buprenorphine. Such an observation of increasing opioid use in a teaching hospital highlights the need for further investigations into whether similar trends can also be found in other large surgical units and academic centers. Moreover, studies with detailed patient-level data including pre- and postoperative opioid prescription rates are needed to investigate better these trends and whether in-hospital pain management policies should be based on the standardized guidelines. Meanwhile, monitoring the rationality of in-hospital opioid use, especially in the bed wards, may be recommended in light of our findings.

## Supplementary Information

Below is the link to the electronic supplementary material.

Supplementary file1 (PDF 310 KB)

## Data Availability

The datasets generated and analyzed during the study are not publicly available, and the authors do not have permission to share the data. The access to used dataset and material needs to be requested from the local institutional review board of Helsinki University Hospital. More information can be inquired from the corresponding author.
