# Supplementary material for: In-hospital postoperative opioid use and its trends in neurosurgery between 2007 and 2018
Source: Acta Neurochir (Wien). 2021 Oct 18;164(1):107–16. doi: 10.1007/s00701-021-05021-9 (PMC8761135; doi:10.1007/s00701-021-05021-9)
Supplement: Supplementary file 1 — Supplementary file1 (PDF 310 KB) [file 701_2021_5021_MOESM1_ESM.pdf]

In-hospital postoperative opioid use and its trends in neurosurgery between 2007 and 2018

Ilari Rautalin, MD[1]; Miia Kallio, MSc[2]; Miikka Korja MD, PhD[1]

[1]Department of Neurosurgery, University of Helsinki and Helsinki University Hospital, P.O. Box 266, FI-00029 Helsinki, Finland

[2]HUS Pharmacy, Hospital Pharmacy of Helsinki University Hospital (HUS), P.O. Box 440, FI-00029 Helsinki, Finland

Correspondence to Ilari Rautalin, Department of Neurosurgery, University of Helsinki, P.O. Box 266, FI-00029 Helsinki, Finland; E-mail address: ilari.rautalin@helsinki.fi; Telephone: +358 947187604; Fax: +358 947187616; ORCID 0000-0002-6283-0398

Supplementary Information

Online Resource 1. Defined daily doses (DDDs) for consumed opioids and their administration routes.

Opioid (generic name) and route of administration	ATC code	DDD
Alfentanil<br>• Injection	N01AH02	 1.2 mg*
Buprenorphine (pain)<br>• Injection<br>• Sublingual<br>• Transdermal	N02AE01	 1.2 mg<br>1.2 mg<br>1.2 mg
Buprenorphine (opioid dependency)<br>• Sublingual	N07BC01	8 mg
Codeine + paracetamol<br>• Oral	N02AJ06	 3 tablets**
Fentanyl<br>• Injection<br>• Transdermal	 N01AH01<br>N02AB03	 1.2 mg*<br>1.2 mg
Hydromorphone<br>• Oral	N02AA03	 20 mg
Methadone<br>• Oral	N07BC02	 25 mg
Morphine	N02AA01	

• Injection<br>• Oral		30 mg<br>100 mg
Oxycodone (oxycodone + naloxone)<br>• Injection<br>• Oral	N02AA05 (N02AA55)	 30 mg<br>75 mg
Pethidine<br>• Injection	N02AB02	 400 mg
Tramadol<br>• Injection<br>• Oral	N02AX02	 300 mg<br>300 mg

*As specific DDDs have not been defined for general anesthesia drugs (i.e. intravenous fentanyl and alfentanil in our case), we calculated the DDDs for these products based on their relative effects compared to oral morphine and transdermally administered fentanyl

** Since the DDD for combined products are established by the number of items, the yearly consumption of combination products (codeine and paracetamol) was defined by the number of issued tablets.

Online Resource 2. Number of treatment days in the neurosurgical study unit between 2007 and 2018

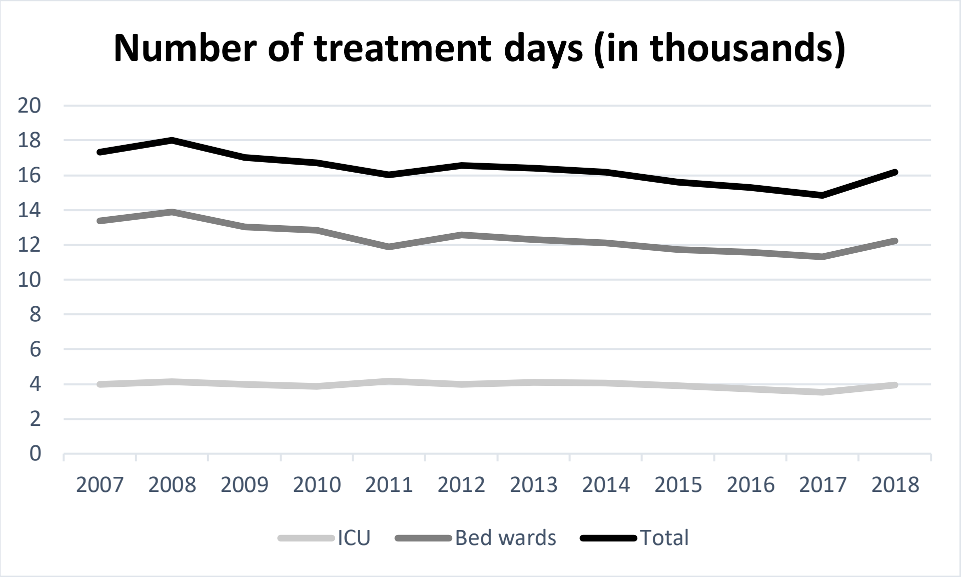

Online Resource 3. Case mix and its time trends between 2012 and 2018.

	Number of operations 2012–2018 (% of all)	Number of operations 2012–2014 (% of all)	Number of operations 2016–2018 (% of all)	Change between 2012–2014 and 2016–2018	Annual change (95% CIs), p-value for linearity
Spinal fusions	3958 (17)	1766 (18)	1646 (17)	-7%	0% (-6 – 5) p=0.90
Tumor surgeries	3013 (13)	1442 (14)	1120 (11)	-22%	-6% (-3 – -8), p < 0.001
Spinal decompressions	2351 (10)	923 (9)	1080 (11)	+17%	4% (2 – 6), p=0.002
CSF-related surgeries	2291 (10)	933 (9)	1039 (10)	+11%	3% (-1 – 8), p=0.14
Trepanations	2216 (10)	912 (9)	988 (10)	+8%	1% (-1 – 4), p=0.20
Cerebrovascular surgeries	1362 (6)	804 (8)	406 (4)	-50%	-15% (-10 – -19), p < 0.001
TBI- and SICH-related surgeries	1035 (4)	464 (5)	407 (4)	-12%	-3% (5 – -10), p=0.41
Spinal discectomies	847 (4)	334 (3)	393 (4)	+18%	4% (0 – 8), p=0.04
Endovascular operations	759 (3)	172 (2)	487 (5)	+183%	27% (19 – 37), p < 0.001
Stereotactic operations	569 (2)	207 (2)	275 (3)	+33%	8% (-1 – 18), p=0.07
Other operations	4757 (21)	2007 (20)	2087 (21)	+4%	1% (-1 – 4), p=0.19
All operations	23 158 (100)	9964 (100)	9928 (100)	-0.3%	0% (-1 – 1) p=0.57

CI=confidence interval; CSF=cerebrospinal fluid; SICH=spontaneous intracranial hemorrhage; TBI=traumatic brain injury

Online Resource 4. Proportions of injected, oral and transdermal opioid consumption in the ICU and bed wards. Values are adjusted by DDDs.

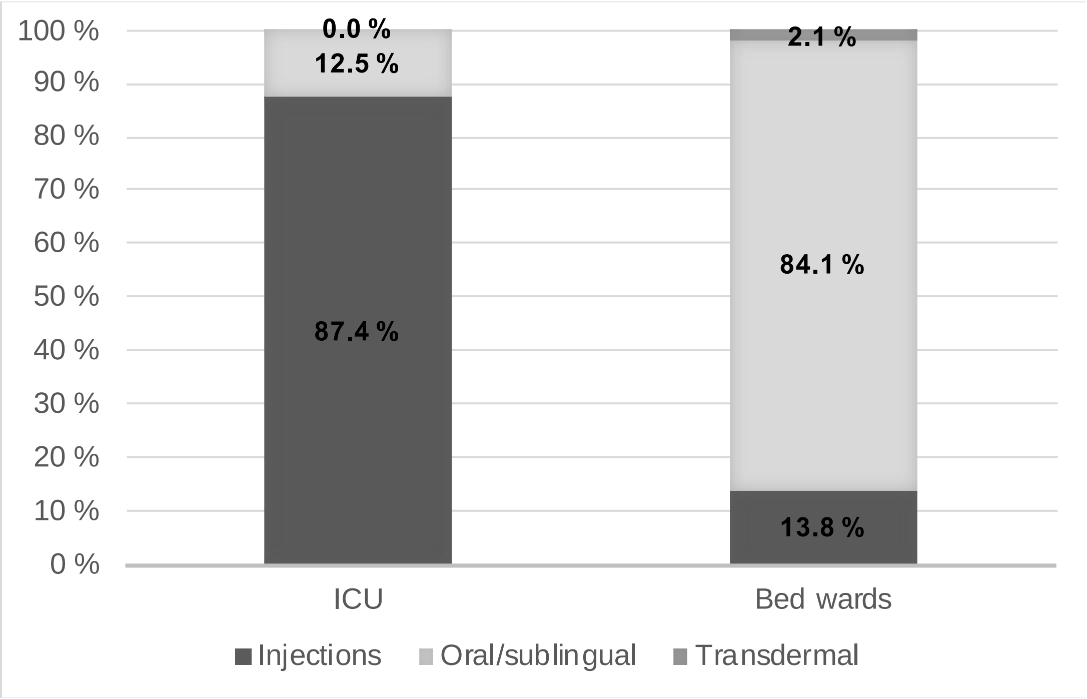

Online Resource 5. Trends in opioid use by route of administration in the ICU (black lines) and bed wards (grey lines). The Y-axis describes adjusted yearly consumption rates (DDDs/100 bed days). Solid lines indicate fitted regression values; dashed lines indicate 95% confidence intervals.

a) injected opioids

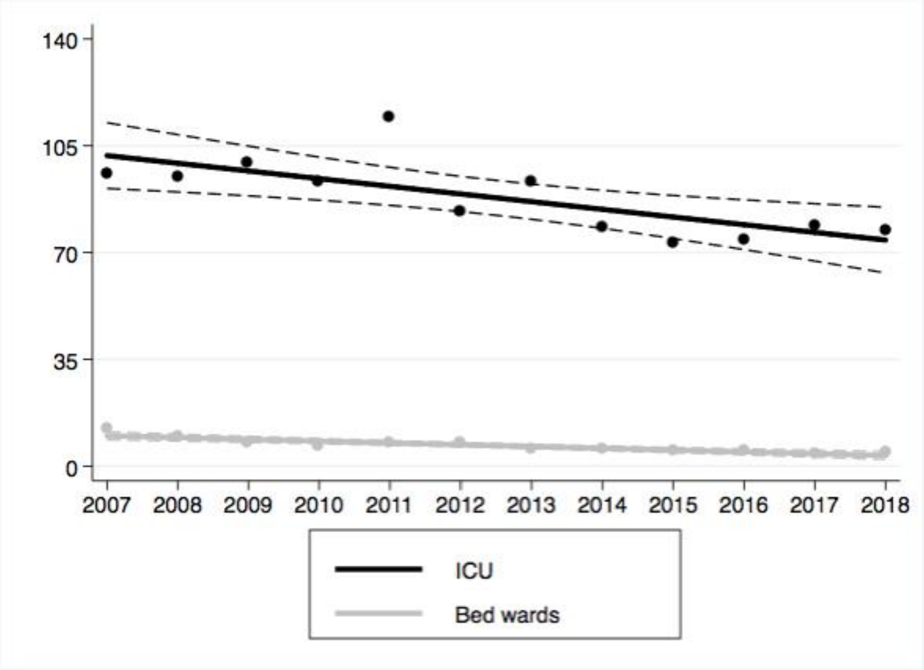

b) oral/sublingual opioids. Since the true consumption of oral/sublingual opioids ended almost completely in 2011 in the ICU (black dots), the fitted regression line (illustrating the trend of consumption) has negative values onwards 2015.

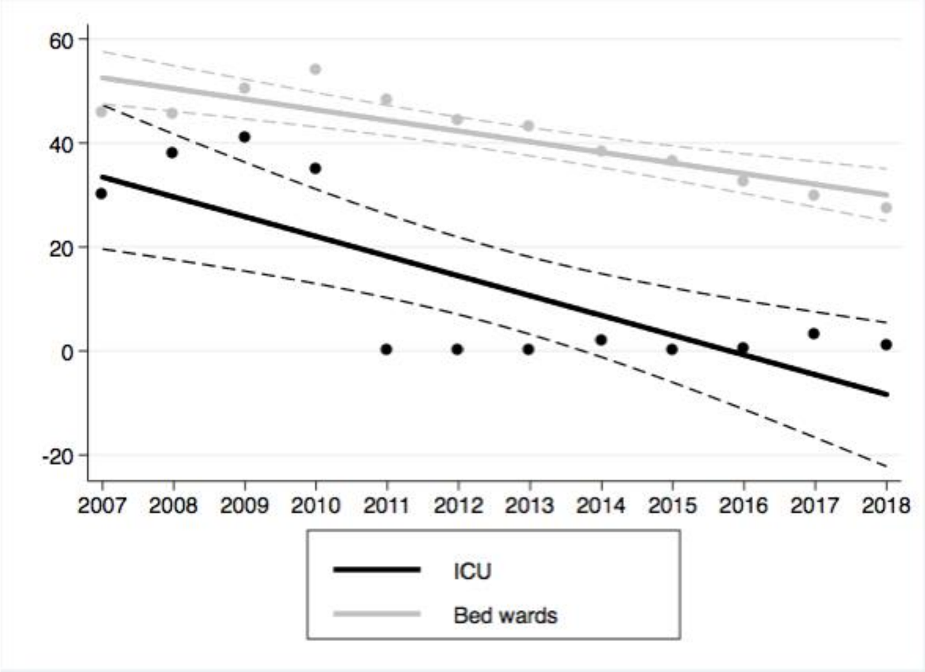

c) transdermal opioids

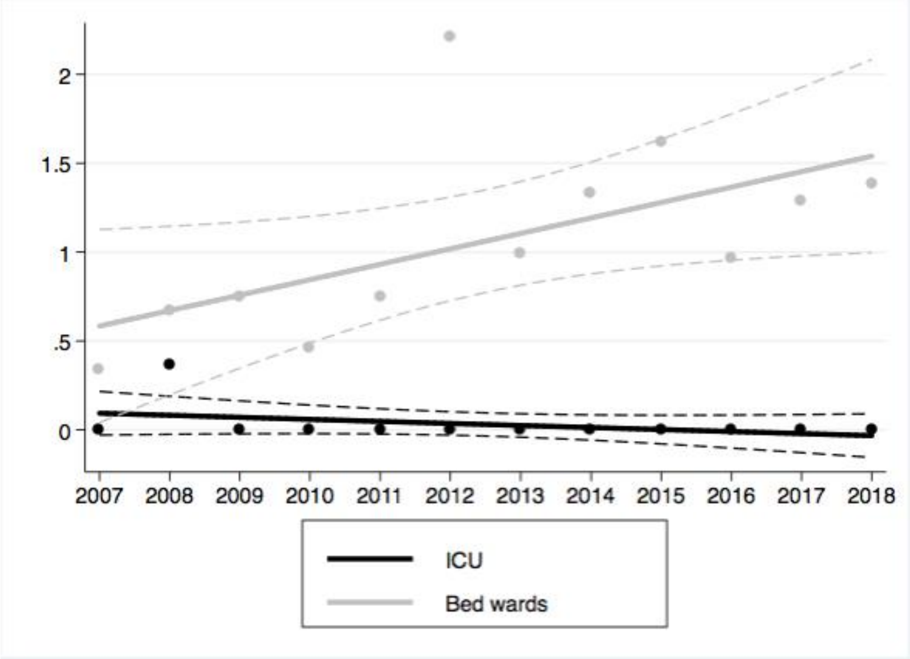

Online Resource 6. Nationwide opioid analgesic use (DDDs/1000 individual/day) in Finland in 2007 and 2018. Data is extracted from the Finnish Statistics of Medicines, versions 2009 and 2018.

Opioid	Consumption in 2007 (DDD/1000 individual/day)	Consumption in 2018 (DDD/1000 individual/day)	Change in %
Overall (all under ATC-code of N02A)	16.09	13.92	-13.5%
Weak opioids*	12.87	9.08	-29.4%
Strong opioids**	2.97	4.51	+51.9%
Buprenorphine	0.15	1.38	+820.0%
Codeine (+ non-opioid analgesic)	9.98	6.46	-35%
Fentanyl	1.40	0.83	-40.7%
Hydromorphone	NR	0.05	NA
Morphine	0.22	0.29	+31.8%
Oxycodone	1.20	1.96	+63.3%
Pethidine	NR	NR	NA
Tramadol	2.89	2.62	-9.3%

*weak opioids = codeine (+ non-opioid analgesic) and tramadol

**strong opioids = buprenorphine, fentanyl, hydromorphone, morphine and oxycodone

Online Resource 7. Overall opioid consumption between the two bed wards of the neurosurgical unit.

	Overall DDDs per 100 treatment days between 2007 and 2018 (% of total consumption on bed wards)	
Opioid	Regular bed ward	Bed ward with step-down unit
Overall opioid use		
• Overall	57.9 (58)	42.4 (42)
• Injection	6.0 (44)	7.5 (56)
• Oral	51.0 (60)	33.7 (40)
• Transdermal	0.8 (40)	1.2 (60)
Weak opioids	42.6 (64)	23.7 (36)
Strong opioids	15.3 (45)	18.6 (55)
Alfentanil		
• Injection/overall	0 (0)	0.005 (100)
Buprenorphine		
• Overall	0.8 (47)	0.9 (53)
• Injection	0 (0)	0.02 (100)
• Sublingual	0.5 (50)	0.5 (50)
• Transdermal	0.3 (43)	0.4 (57)
Codeine (+paracetamol)		
• Oral/overall	35.1 (63)	20.6 (37)
Fentanyl		
• Overall	0.5 (38)	0.8 (62)
• Injection	0.007 (88)	0.001 (12)

• Transdermal	0.5 (38)	0.8 (62)
Hydromorphone<br>• Oral/overall	 0.04 (29)	 0.1 (71)
Methadone<br>• Oral/overall	 1.0 (67)	 0.5 (33)
Morphine<br>• Overall<br>• Injection<br>• Oral	 0.3 (23)<br>0.1 (11)<br>0.2 (50)	 1.0 (77)<br>0.8 (89)<br>0.2 (50)
Oxycodone<br>• Overall<br>• Injection<br>• Oral	 12.7 (45)<br>5.9 (47)<br>6.8 (44)	 15.3 (55)<br>6.6 (53)<br>8.7 (56)
Pethidine<br>• Injection/overall	 0.004 (40)	 0.006 (60)
Tramadol<br>• Overall<br>• Injection<br>• Oral	 7.5 (71)<br>0.04 (40)<br>7.5 (71)	 3.1 (29)<br>0.06 (60)<br>3.1 (29)
